# Diseases of the Stomach and Upper Alimentary Tract

**Published:** 1911-04

**Authors:** 


**Diseases of the Stomach and Upper Alimentary Tract.** By **Anthony Bassler,** M.D., Visiting Gastroenterologist to the People's Hospital, and Visiting Physician to St. Mark's Hospital Clinic, New York. Octavo. pp. 836. Illustrated. Philadelphia: F. A. Davis Co. 1910. (Cloth, $6.00.)

In this new work of Bassler's he states that as considerable difference of opinion exists among the foremost gastroenterologists on many points which have to do with the successful treatment of the diseases in question, he has elected to follow along the lines of his own making, warranted by his long experience in the observation and treatment of diseases of the gastrointestinal tract. Since surgery has begun to take such an active part in the treatment of diseases of the stomach and intestines it devolves upon the non-operating physician, who is usually consulted first, to take every means to clear up in his own mind as far as may be possible all doubts as to the actual condition present, in order that he may clearly recognise the circumstances which justify surgical interference or contraindicate such a procedure.

In discussing the various methods of treating acute ulcer of the stomach the author strongly advises the use of belladonna as a drug of the greatest value. His conclusions are: "Belladonna effectively and positively controls excessive secretion and mobility thereby favoring repair of the ulcer; it holds in check the worked up stomach secretion; it has a better effect on the relief of symptoms of pain, vomiting, hemorrhage, and local distress, than bismuth, silver nitrate, morphine, or the alkalies, single or combined; it is easy to administer, does not impair digestion, nor does it cause distention." Throughout the entire volume the recommendations are logical and sound. The author has accomplished his purpose admirably and the book will be welcomed by the medical profession. The illustrations are excellent, and the mechanical part all that need be desired. J. A.R.

